# FloatingBlue: A Delay Tolerant Networks-Enabled Internet of Things Architecture for Remote Areas Combining Data Mules and Low Power Communications

**DOI:** 10.3390/s24196218

**Published:** 2024-09-26

**Authors:** Ruan C. M. Teixeira, Celso B. Carvalho, Carlos T. Calafate, Edjair Mota, Rubens A. Fernandes, Andre L. Printes, Lennon B. F. Nascimento

**Affiliations:** 1 Postgraduate Program in Electrical Engineering, Federal University of Amazonas, Manaus 69080-900, Brazil; ccarvalho_@ufam.edu.br; 2 Embedded Systems Laboratory, State University of Amazonas, Manaus 69050-020, Brazil; rubens.eng.elet@gmail.com (R.A.F.); aprintes@uea.edu.br (A.L.P.); lennonbfn@gmail.com (L.B.F.N.); 3 Universitat Politècnica de València, 46022 València, Spain; calafate@disca.upv.es; 4 Institute of Computing, Federal University of Amazonas, Manaus 69080-900, Brazil; edjair@icomp.ufam.edu.br

**Keywords:** IoT, DTN, BLE, UAV, low power communications

## Abstract

Monitoring vast and remote areas like forests using Wireless Sensor Networks (WSNs) presents significant challenges, such as limited energy resources and signal attenuation over long distances due to natural obstacles. Traditional solutions often require extensive infrastructure, which is impractical in such environments. To address these limitations, we introduce the “FloatingBlue” architecture. This architecture, known for its superior energy efficiency, combines Bluetooth Low Energy (BLE) technology with Delay Tolerant Networks (DTN) and data mules. It leverages BLE’s low power consumption for energy-efficient sensor broadcasts while utilizing DTN-enabled data mules to collect data from dispersed sensors without constant network connectivity. Deployed in a remote agricultural area in the Amazon region, “FloatingBlue” demonstrated significant improvements in energy efficiency and communication range, with a high Packet Delivery Ratio (PDR). The developed BLE beacon sensor achieved state-of-the-art energy consumption levels, using only 2.25 µJ in sleep mode and 11.8 µJ in transmission mode. Our results highlight “FloatingBlue” as a robust, low-power solution for remote monitoring in challenging environments, offering an energy-efficient and scalable alternative to traditional WSN approaches.

## 1. Introduction

Wireless Sensor Networks (WSNs) are advantageous for monitoring vast and remote areas such as forests, primarily due to the lower operational costs of installation and maintenance [1]. However, these scenarios pose significant challenges for WSNs, such as energy constraints of sensor nodes, typically battery-powered, and the requirement for long-distance communication to cover extensive areas [2]. Recent studies, such as [3], highlight the importance of addressing these challenges by optimizing resource allocation strategies to enhance the performance and efficiency of WSNs, particularly in scenarios where real-time information timeliness is critical.

Monitoring vast and remote areas (such as forests) using WSNs presents challenges, such as energy limitations and signal attenuation due to natural obstacles. Traditional solutions require extensive infrastructure, which is impractical in such isolated environments. Therefore, there is a need for architectures that can overcome these obstacles, especially in regions like the Amazon, where network coverage and energy efficiency are essential for the success of environmental monitoring operations.

Low-Power Wide-Area Networks (LPWAN) enable long-range communication with low energy consumption. However, they face limitations in forested areas due to natural obstacles like trees and leaves, which can significantly reduce signal range. For instance, in an open environment, LPWANs can reach up to 15 km; however, in densely forested areas, this range may be reduced to only 1–2 km [4]. Additionally, LPWANs require infrastructure, including installing gateways with a continuous electricity supply and connectivity to the Internet for transmitting the collected data [5].

Delay and Disruption Tolerant Networks (DTNs) can handle network interruptions, operating effectively in environments with high latencies and disconnections. However, DTNs require superior processing capability and energy consumption from nodes, which can be impractical for battery-powered devices like sensors [6,7,8]. To mitigate or eliminate the need for network infrastructure and to address the challenge of long-distance communications, different studies propose the adoption of mobile communication nodes known as data mules [9,10], relay nodes [6,11], or sink nodes [12]. These nodes collect sensor data on the ground and forward it to a gateway, processing, control station, or another forwarding node.

A promising strategy in this area involves using data mules through Unmanned Aerial Vehicles (UAVs) that can fly over remote areas, gather sensor data, and transmit it to destinations, mitigating disconnection issues and reducing ground infrastructure [13,14].

In this context, we present the “FloatingBlue” architecture, which combines Bluetooth Low Energy (BLE) technology with Delay Tolerant Networks (DTN) and data mules. The “FloatingBlue” stands out for its innovation by uniquely integrating these components, providing a monitoring solution that does not depend on constant network connectivity. This approach enables the efficient collection of data from dispersed sensors, leveraging BLE’s low energy consumption and the robustness of DTN in scenarios of frequent disconnection [15].

Furthermore, a BLE node can provide a lower energy consumption alternative through its broadcast mode, where the protocol allows communication without the need for a connection to transmit packets, further optimizing energy consumption [16,17]. In this study, we also analyze the influence of energy consumption by embedded software in BLE devices, comparing different firmwares based on bare-metal programming [18]. In particular, we investigate the development of low-level software to control the hardware of a BLE sensor directly, compared to devices for which we developed software using Real-Time Operating System (RTOS).

Thus, in this article, we propose the practical development of a new wireless sensor network architecture called “FloatingBlue”, and the main contributions of this work are:

1. Innovative architecture: development of a new WSN architecture that expands network coverage through data mules communicating via DTN, while reducing energy consumption at terminal nodes by utilizing an optimized BLE protocol.
2. Real-world validation: practical implementation and extensive testing in a real-world scenario in the Amazon, demonstrating the viability and efficiency of the “FloatingBlue” architecture in adverse environments.
3. Energy consumption optimization: development of optimized firmware for BLE devices, resulting in significantly lower energy consumption than traditional approaches, which is crucial for extending the lifespan of sensors in remote areas.

The remainder of this paper is organized as follows: In the next section, we provide an overview of related studies on this topic. Our proposed “FloatingBlue” architecture is presented in Section 3. Then, in Section 4, we provide implementation details, including hardware and software. Section 5 presents the different experimental results obtained regarding energy consumption, functional experiments and communication experiments, including discussion. Finally, Section 6 presents the main findings of our work, along with future research lines.

## 2. Related Studies

In the context of WSN for applications in rural and remote environments, LPWAN technologies have demonstrated particular promise due to their capacity for long-range communication and the low energy consumption of their transceivers [5,19]. When solely considering the aspect of sensor energy consumption, the BLE technology, particularly when used in the broadcasting communication mode, emerged as a promising alternative due to its low energy consumption. This was due to the minimal computational demand required for the transmission of broadcasting messages, which are known as advertisements [20]. Such devices were designated beacons. They were widely adopted in a variety of applications, including health monitoring for disease prevention [21,22] and workplace safety monitoring through Personal Protective Equipment (PPE) equipped with beacons [16].

In comparison, in idle mode, BLE demonstrated energy efficiency similar to or superior to certain LPWAN technologies. The study by Schrader et al. [23], which utilized BLE beacons, reported results of 5.9 µA in sleep mode, while Aguilar et al. [24] cited a consumption of 1.19 µA. However, it was observed that WSN solutions utilizing a secondary wake-up radio exhibited higher energy consumption, with levels reaching 270 nW, as discussed in reference to the literature [25]. This technology operated in conjunction with the primary transceiver, employing a low-power radio system designed to activate the device from its low-power radio system to transition the device from a low-power state to an operational mode. Despite the low consumption, implementation of a Wake-up Radio (WuR) introduced additional complexities to the device, requiring two radios and often two antennas. Furthermore, the range of WuR was typically limited to, between 5 and 30 m [26]. In contrast, BLE offered advantages such as simplicity of implementation and a more extended transmission range, making it preferable in various applications.

Despite the highlighted advantages of BLE, studies such as those in [23,24] were conducted in controlled environments, which may need to reflect real-world field conditions accurately. These limitations included the lack of testing in scenarios with varied environmental interference, which could impact BLE devices’ energy performance and communication range.

A strategy for device mobility scenarios is DTN, which enables data transmission regardless of an instantaneous end-to-end path between source and destination. These networks can operate effectively in situations with significant latencies and network interruptions between nodes [27,28].

In addition, many studies suggested adopting DTNs using data mules based on UAVs [13,14]. UAVs have rapidly expanded across various applications, from precision agriculture to environmental monitoring in sensitive ecosystems. In [29], the authors examined the effects and risks of digital technologies in pasture monitoring, using UAVs, sensors, and data communication networks to manage cattle and sheep. Similarly, [30] explored computer vision for detecting hares and roe deer through aerial drone images. These approaches demonstrated the potential of UAVs for monitoring different ecosystems and helping mitigate impacts on natural habitats, particularly in ecologically sensitive areas. For instance, in [31], the application of Deep Learning techniques to identify weeds and monitor crop health in rice fields using low-altitude UAVs showcased the efficiency of these technologies with minimal computational resources. Nevertheless, studies like [30,31] also raised concerns about potential interference with wildlife and ecosystem degradation.

Despite DTN being efficient in data mule application contexts, it was not beneficial to use DTN technologies in low-power end nodes in WSNs. This was because end nodes would have required hardware with significant processing power and high energy consumption to function as DTN nodes [6,7,8].

DTNs offered a robust solution for data transfer in extreme conditions such as environmental disaster scenarios, where the native network infrastructure of the location had been affected, and inhospitable environments, as discussed in [6,7,11,13,32,33]. For example, Solpico et al. [13] proposed using DTN to assist firefighting teams in environmental disaster scenarios. Another implementation of DTN in challenging environments was discussed in [34], which stood out for creating a DTN-based WSN for ecological monitoring in the Antarctic continent. Additionally, Jeon et al. [33] explored the use of DTNs in a subaquatic context, proposing a network architecture for sensors operating under high-latency conditions typical of underwater communication.

Furthermore, DTNs demonstrated outstanding agricultural sector and environmental monitoring potential. In [8], the authors proposed an architecture for WSNs employing DTNs for monitoring environmental parameters and providing geopositioning without relying on traditional network infrastructure. In agriculture, Ayele et al. [35] proposed using DTN nodes on agricultural tractors to collect data from static sensors dispersed across large properties.

Opportunistic Networks (OppNets) are a type of DTN characterized by inheriting the exact mechanisms that ensure communication between nodes subject to disconnections (store-carry-forward). Still, they are not limited to the TCP/IP stack [36,37]. In [17], the authors developed an opportunistic network using collars equipped with BLE for monitoring the physiological activities of wild animals in remote and extensive habitats. In this setup, the animals wearing the collars acted as data mules, facilitating the transport and transmission of information as they moved through the environment. This approach enabled efficient large-scale monitoring without the need for fixed infrastructure, leveraging the natural mobility of animals to optimize data collection. Additionally, in the context of OppNets, Tsai and Chen [38] proposed a strategy to minimize energy consumption in opportunistic networks; in particular, they used BLE advertising messages running on the background of smartphones as data mules. This technique significantly reduced energy demand by maintaining communication at low consumption levels.

Some studies in the literature presented architectures that used BLE-based WSNs for communication with data mules equipped with DTN. However, only some studies integrated all these elements. Ochiai et al. [35] presented simulated models employing the BLE/DTN/data mule architecture, but they needed to conduct practical experiments that encompassed the proposal made in the current study.

Table 1 summarizes the scientific literature studies similar to the proposed WSN architecture that adopted data mules in the WSN architecture, exploring communication consumption or range as a result. The table also presents some properties used in the research, such as the adoption of DTN, including OppNet, and the technology used for the end node of the WSN architecture.

## 3. FloatingBlue

“FloatingBlue” is a WSN communication architecture in which the main elements are Data Mules (DMs) that communicate among themselves via DTN and with sensor nodes using the BLE protocol in broadcasting mode.

Sensing is challenging in large plantations and forested areas, such as those in the Amazonian jungle, due to the distances involved. Many sensors are battery-powered, and frequent replacement in remote locations can be impractical or costly. Energy-efficient technologies like BLE are crucial for extending sensor lifespans, especially in applications with sporadic data transmission. Therefore, the need for an architecture like “FloatingBlue” becomes evident as it optimizes sensor coverage and energy efficiency in challenging environments.

Continuous connectivity is a challenge in remote locations with adverse environmental conditions. Connectivity can often be intermittent, making real-time data transmission difficult. DTN offers solutions to these situations by enabling data transmission in high-latency and frequently disconnected scenarios. Moreover, deploying communications infrastructure in remote areas can be relatively inexpensive. “FloatingBlue” proposes a solution that minimizes the need for fixed infrastructure by relying on data mules to transport information from sensors to a central station.

The “FloatingBlue” architecture was explicitly developed to address these challenges. Using drones equipped with DTN as data mules and sensors that communicate with these drones via BLE, “FloatingBlue” provides broader coverage and energy-efficient transmission even in adverse conditions. This combination enables farmers and environmental managers to obtain monitoring data without needing extensive and costly network infrastructure.

**Figure 1:**
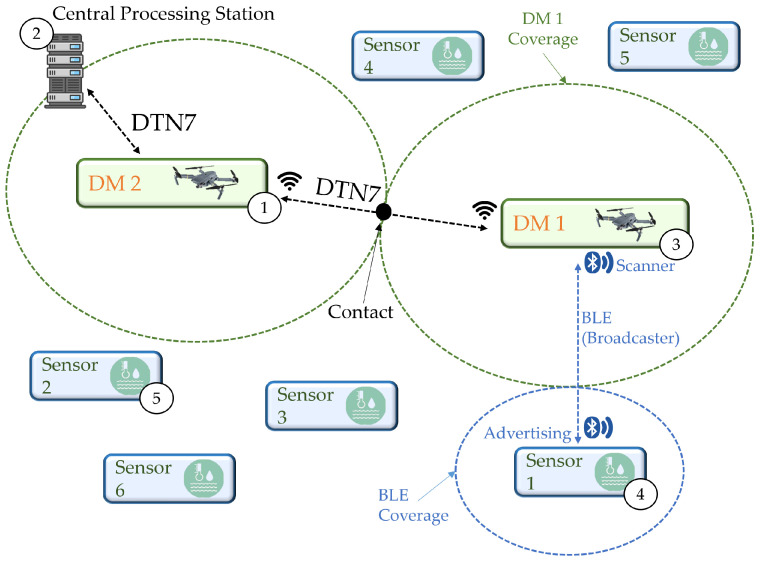
Proposal of the WSN FloatingBlue architecture.

Figure 1 illustrates the proposed architecture. DM1 (3) is a mobile microcontroller device that can be attached to a vehicle, manned or unmanned, depending on the application, with each DM responsible for a sub-network. A sub-network consists of DMs and static SN, such as (4, 5). DM1 follows a movement routine to capture information from SNs as they enter their communication range. The data from SNs are transmitted and stored in DM1, then forwarded to other DM nodes (1) until they are offloaded at a central processing station (2).

**Figure 2:**
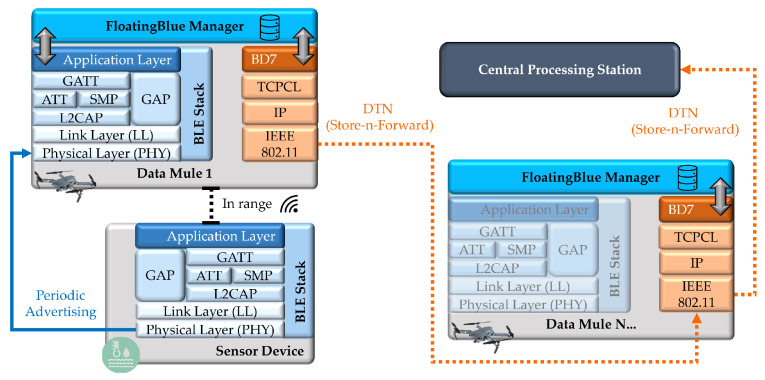
Protocol stacks defined for the FloatingBlue architecture.

Each DM performs periodic scanning routines, while the SN transmits advertising packets with data collected by the integrated sensors. The DM nodes communicate with each other using Bundle Protocol 7, and, at the lower layers, TCP/IP (Transmission Control Protocol Convergence-Layer-TCPCL [46] and IPv6/IPv4) and IEEE 802.11 are used. Figure 2 illustrates the protocol layers adopted in the “FloatingBlue” architecture.

The DM node operates in two modes: collection mode and forwarding mode. In the collection mode, the DM implements both protocols, using BLE to receive data transmitted by SNs, and DTN Protocol 7 to encode the data into the bundle format for transmission to the destination entity. In the forwarding node mode, the node implements Bundle Protocol 7, performing store-and-forward mechanisms for the bundles.

**Figure 3:**
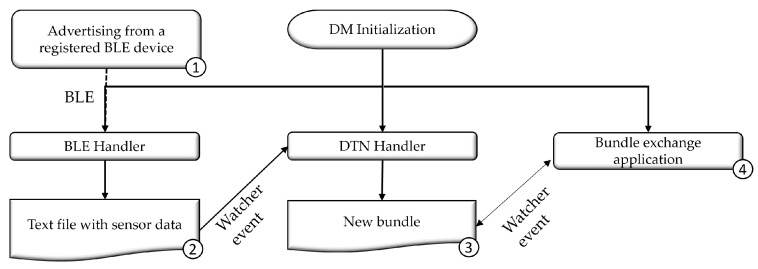
Flowchart of the “FloatingBlue Manager”.

In the application layer of the protocol stack, software services form the “FloatingBlue Manager” layer, as shown in Figure 2. Two services are executed in this layer: “BLE Handler” and “DTN Handler”. These applications integrate the DTN Protocol 7 and the BLE protocol stacks. The interaction dynamics and message flow between these services in the application layer are further detailed in Figure 3.

The cycle begins with receiving an advertising signal (1) from a previously registered SN by the DM. When a registered SN is detected, the *BLE Handler* service collects the data, formats it, and saves it into a text file (2). Subsequently, the *DTN Handler* service transforms this file into a bundle (3), preparing it for transmission to the destination node (4).

## 4. Implementation

This section will detail how our proposed architecture was implemented using real devices. First, we will describe the different hardware components used, and then we will explain the software configuration adopted.

### 4.1. Hardware

**Figure 4:**
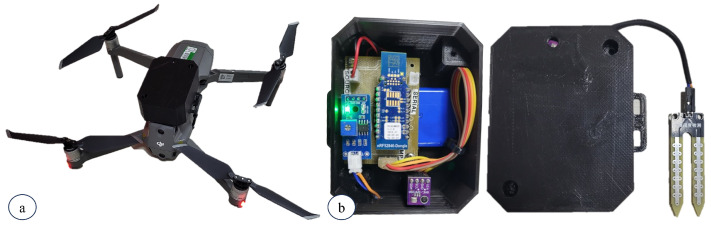
Implementation of DM hardware using UAV (**a**) and SN with NRF52840 beacon (**b**).

The DM hardware was developed using a Raspberry Pi 3 Model B, installed in a vehicle. The Raspberry Pi 3 features Bluetooth 4.1 and IEEE 802.11ac communication capabilities, allowing BLE and DTN protocols over Wi-Fi in the proposed architecture, respectively. Figure 4a depicts our final DM prototype based on a commercial UAV.

The sensor node was designed to function as an environmental monitoring device. The device was implemented using a BLE beacon nRF52840 dongle from Nordic Semiconductor, a BME680 gas sensor, and a soil moisture sensor, as illustrated in Figure 4b.

### 4.2. Embedded Software

The applications of the “FloatingBlue” manager on the DM are background software services. The implementation of Bundle Protocol 7 adopted is based on the dtn7 library in the Go language (dtn7-go). In the embedded software, a library initialization service is responsible for implementing a bundle node in addition to the *BLE Handler* and *DTN Handler* services.

**Figure 5:**
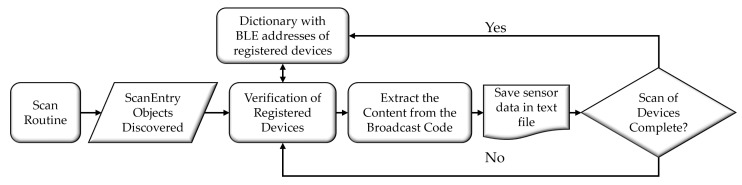
Flowchart of the *BLE Handler* algorithm.

The *BLE Handler* software service collects parameters the SNs measure. Figure 5 illustrates the flowchart of the algorithm. The application maintains a Public Device Addresses (PDA) list of pre-registered BLE-enabled devices. The service periodically performs scanning routines to identify BLE devices within the DMs range. This scanning occurs in loops with defined intervals to maximize detection chances. When a device is detected, the data it collects is compared with the PDAs registered in the DM. If the service finds the address of a registered node, it extracts the Broadcast Code (0x2D 2D) from the advertising, along with the corresponding PDA and the timestamp of the collection. This process allows for the distinction between SNs and enables recording the exact instant of data collection. The collected data are then stored in a file, typically a text file by default.

The flowchart in Figure 5 is designed to reflect the logical sequence of operations performed by the *BLE Handler* algorithm. The process begins with a scanning routine aimed at discovering nearby BLE-enabled devices. Upon finding a device, the algorithm verifies if it is among the pre-registered devices by comparing the detected Public Device Address (PDA) with those stored in the dictionary. This step is important for filtering out irrelevant devices and focusing only on those of interest. Once a registered device is identified, the flowchart outlines the extraction of the Broadcast Code and the corresponding PDA, then stores these data, including the timestamp, into a text file. This design ensures the data collection is systematic and traceable, which is essential for subsequent analysis. The decision block at the end of the flowchart determines whether the scanning process should continue or terminate, depending on whether all devices have been scanned. This loop maximizes detection accuracy and ensures comprehensive data collection within the *BLE Handler*’s operational range.

**Figure 6:**
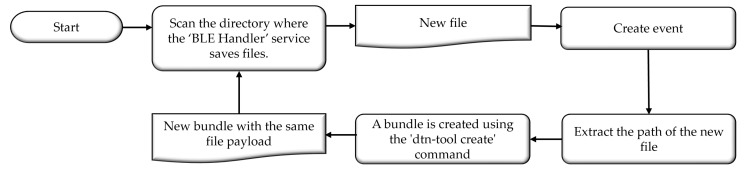
Flowchart of the *DTN Handler* algorithm.

The *DTN Handler* software service, as illustrated in Figure 6, is responsible for transforming the output file from the *BLE Handler* service into a bundle. The service consists of a watcher application that monitors the directory where the output files from the *BLE Handler* are saved. Upon the creation event of a new file, the watcher calls the create tool from dtn7. This tool receives the path of the new file and implements an Application Agent (AA) that transforms the file’s payload into a bundle using the Bundle Protocol Agent.

In parallel to the services, a bundle exchange tool is activated. The tool monitors the bundle directory using a watcher module in the Go language. Upon detecting the presence of a new bundle file, the tool sends the bundle to the AA via a WebSocket API. The AA forwards the bundle to the CLA, which transmits it to the destination node using the TCPCL transport layer.

The flowchart in Figure 6 is designed to represent the steps involved in converting the output files from the *BLE Handler* into DTN bundles. The process starts with a continuous scan of the directory where the *BLE Handler* saves files. When a new file is detected, the system creates an event to trigger the bundle creation process. The flowchart then outlines how the file path is extracted and passed to the “dtn-tool create” command, which generates a new bundle with the same file payload. This design ensures that every new file is promptly and systematically converted into a DTN bundle, ready for transmission. The linear and sequential structure of the flowchart emphasizes the straightforward and automated nature of the process, highlighting how each step directly leads to the next, ensuring the efficient handling of data in the DTN network.

**Figure 7:**
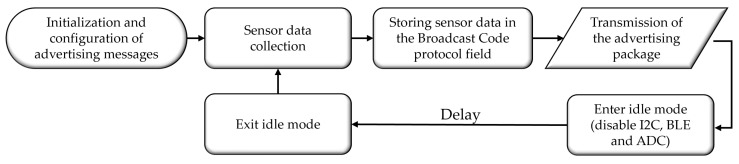
Flowchart of the SN firmware.

The embedded software of the SN consists of a firmware whose main objective is, as shown in Figure 7, to collect data from sensors, format them appropriately, and transmit them via advertising packets. Initially, the software routine establishes the header of the advertising packets. At this stage, the advertising message’s variables are structured, focusing on the broadcast code frame. Additionally, the definition of BLE initialization and transmission interval occurs. Subsequently, data are collected from the BME680 and soil humidity sensors. These data variables are then stored in the Broadcast Code field, followed by a call to update the advertising message function. The data are transmitted only on BLE channel 37. Finally, the BLE radio and peripherals are deactivated, and the sensor is idle to reduce energy consumption. After a programmable interval, the device exits idle mode, activating the deactivated peripherals and returning, in a loop logic, to the data collection step.

The flowchart in Figure 7 is designed to reflect the sequential and cyclical nature of the SN firmware’s operation. The process begins with initializing and configuring advertising messages, ensuring the transmitted data are appropriately formatted and broadcast. The flowchart then moves to the sensor data collection step, where environmental parameters are gathered. These data are subsequently stored in the Broadcast Code protocol field, a step that directly links data collection with transmission. The flowchart also illustrates how, after data are transmitted, the system enters idle mode to conserve energy, disabling key components such as I2C, BLE, and ADC. Including a delay before exiting idle mode and resuming data collection emphasizes the energy-efficient design of the firmware. This structured approach in the flowchart highlights the firmware’s efficiency in handling sensor data while minimizing energy consumption, which is critical for the SN’s performance in field applications.

Two approaches were developed for implementing the SN’s embedded system: one using routines from the Software Development Kit (SDK) v2.3.0 and another with bare-metal firmware, which operates directly on the hardware without requiring an RTOS. This allows for the evaluation of the influence of firmware development methodology on the SN’s energy consumption.

Optimizations were implemented in the bare-metal firmware methodology to reduce the beacon’s power consumption. During idle mode, the high-frequency clock was disabled, and 95% of the System-on-a-Chip (SoC) nRF52840s Random Access Memory (RAM) sectors were deactivated. Additionally, the power supply method of the SoC was optimized. The chip provides two types of regulators, Low-Dropout (LDO) and buck; in the bare-metal firmware, the LDO regulator was disabled. Moreover, compared to the RTOS firmware, the bare-metal firmware’s size was reduced: there was a 99% reduction in RAM usage and a 97% reduction in flash memory usage.

## 5. Results

The “FloatingBlue” proposal was tested in an agricultural environment to verify its effectiveness. During the tests, the overall functionality of the system, as well as the energy consumption and communication range of the SN and associated beacon, were evaluated. These nodes were designed to be energy-efficient in remote areas.

### 5.1. Energy Consumption Analysis Experiments for SNs

To evaluate energy consumption, the Power Profile Kit II from Nordic Semiconductor was utilized to measure the electric current consumed by the SN nodes. Energy consumption tests for the SNs were conducted using a supply voltage of 1.8 V, and an initial transmission power of 0 dBm. The Power Profile Kit II is a high-precision micro-ammeter connected in series with the power source and the SN. The Power Profile Kit II is also connected to a computer via a USB port, and the measurement results are visualized through a graphical interface provided by Nordic Semiconductor.

The energy consumption tests were conducted without the BME-680 and the soil moisture sensor, focusing solely on the BLE beacon’s consumption. This approach compares with related studies focusing similarly on beacon consumption. The results of each test were determined by 60 individual measurements, and then statistical analyses, including the mean and the standard error of the mean, were calculated for the energy consumption in each test scenario.

Comparative energy consumption experiments were conducted to determine the energy consumption of the BLE beacon and the optimized embedded software. These experiments compared the beacon’s energy usage when using firmware routines from the manufacturer’s SDK in an RTOS versus firmware routines in bare-metal. Initially, the beacon’s consumption was measured in idle mode, followed by transmission mode for a 3-byte advertising message, with a transmission power of 0 dBm, and only one active BLE channel (channel 37). Table 2 summarizes the results of these beacon evaluations.

Thus, the optimizations achieved through bare-metal embedded software significantly minimize energy consumption compared to RTOS-based firmware. Therefore, we decided to proceed with the bare-metal firmware for subsequent experiments.

**Figure 8:**
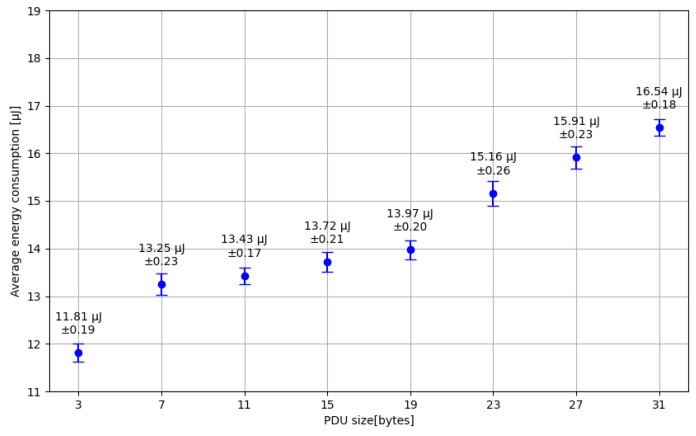
Relationship between energy transmission consumption of the SN and PDU Packet size.

Afterward, energy consumption was analyzed by focusing on the impact of varying the BLE Packet Data Unit (PDU) payload size. It is important to note that the maximum allowed payload size is 31 bytes, divided into 3 bytes for the Flags field and 28 bytes for the actual data. The test was conducted with advertising messages being transmitted at 1-s intervals, inheriting the previous experiment’s transmission power and channel configurations. Figure 8 illustrates the obtained results.

**Figure 9:**
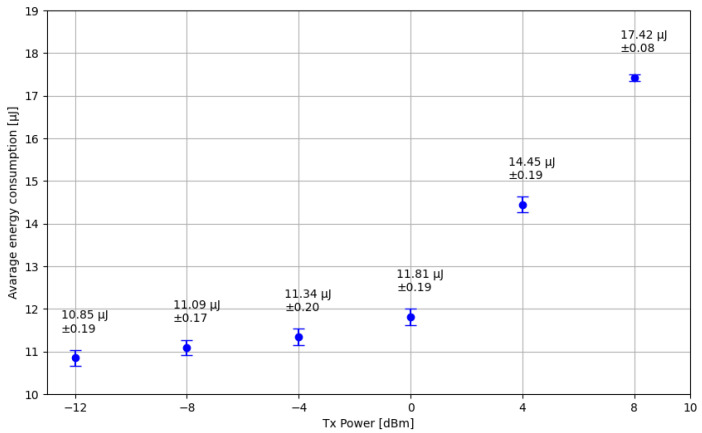
Relationship between energy transmission consumption of the SN transmission power.

Another evaluation of energy consumption conducted, as depicted in Figure 9, involved analyzing the relationship between the transmission power of the SN and its energy consumption. The Nordic nRF52840 SoC allows the BLE transceiver transmission power to be adjusted from −12 dBm to 8 dBm.

**Figure 10:**
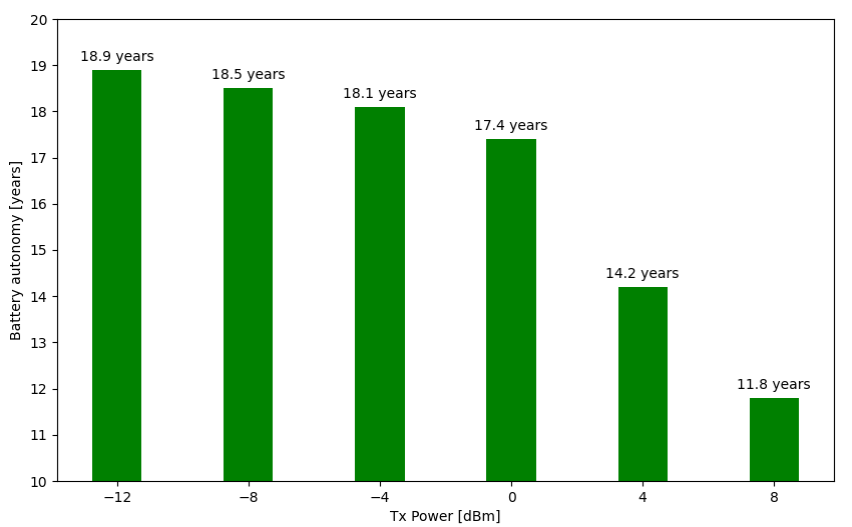
Relationship between battery autonomy of the SN transmission power.

The final energy consumption assessment by “FloatingBlue” architecture is related to the projection of the SN’s battery autonomy concerning the beacon’s transmission power, as depicted in Figure 10. For this, a 1050 mAh and 3.7 V lithium battery was considered, the same specification used in the functional tests of the architecture.

### 5.2. Functional Experiments of the FloatingBlue Architecture

**Figure 11:**
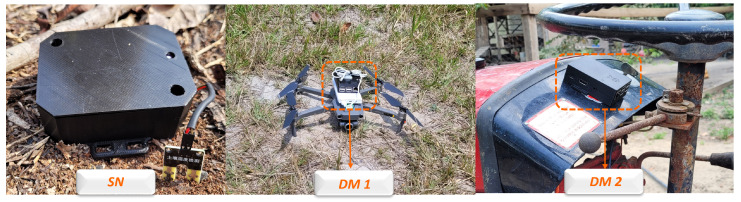
SN and DM installed in the test scenario.

For the functionality tests of the “FloatingBlue” proposal, the SNs were deployed in a citrus plantation in a remote area in the Amazonian jungle. Figure 11 illustrates one of the three SNs placed in the ground and two DMs installed in the test environment. DM 1 was mounted on a DJI Mavic 2 Zoom drone, and DM 2 was installed on a tractor.

Parameters were configured for the BLE and dtn7-go protocols in the DM node. For BLE, the scanner window time was adjusted to 5 s, which is longer than the advertising interval of the SN. The parameters for dtn7 are detailed in Table 3.

During the experiments, a Wi-Fi access point was used to collect results via SSH and establish a WebSocket channel with the DM 2 device. The access point is an optional element in the “FloatingBlue” architecture.

**Figure 12:**
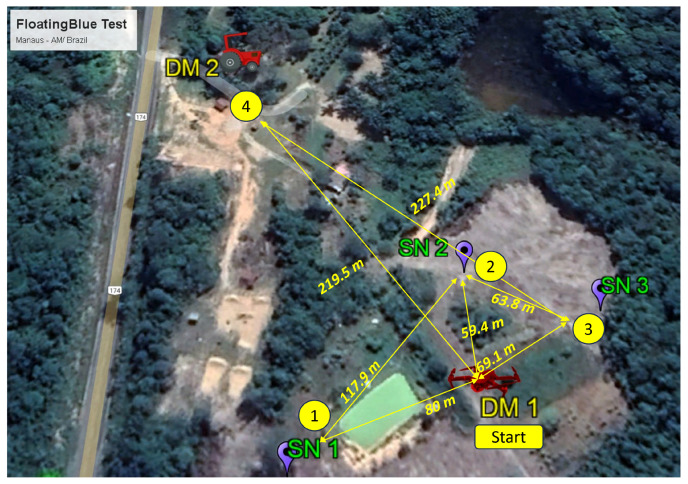
Location of different architecture entities and trajectory of DM 1.

According to Figure 12, the test began with the takeoff of DM 1 from the point labeled “Start”. DM 2 was 219.5 m from the Start point and outside the communication range of DM 1. During the test, the tractor with DM 2 remained stationary, while the UAV carrying DM 1 flew at a constant altitude of 30 m to avoid interference with local vegetation.

After takeoff, DM 1 followed GPS coordinates, sequentially flying over SN 1, SN 2, and SN 3. At each SN, it remained stationary for 3 to 5 min to collect a significant volume of data. It flew over the tractor with DM 2 and returned to the “Start” point. Figure 12 depicts this trajectory. The communication transceiver of the DM 2 was deactivated to ensure disconnection despite the distance of DMs. Later, it was reactivated only when DM 1 directly flew over DM 2. In the end, the data are collected by the processing station, which interfaces with the user once the DM 2 comes within the communication range of the station.

The *DTN Handler* and *BLE Handler* services were analyzed using the ‘dtn show’ tool, which reports the bundle contents, allowing for the analysis of bundle integrity. DM 1 and DM 2 were disconnected, without contact, for 51 min. DM 1 produced 437 bundles collected from the three SNs during this period. When the connection between DM 1 and DM 2 was restored, all bundles from DM 1 were transferred to DM 2 with a Packet Delivery Ratio of 100%.

### 5.3. SN Communication Range Experiments

The communication range between an SN node and a DM was evaluated under line-of-sight conditions without obstructions. To determine the distance between DM 1 (UAV) and a location with known GPS coordinates, which matched the SN node’s location, geopositioning information provided by the UAV’s remote control was used, with 1 m of error.

During the range test, the UAV was maintained at an altitude of 30 m as specified. After reaching this altitude, the UAV was moved horizontally northward, increasing the distance between DM and the fixed SN node until there was no communication between them for a period equal to or greater than 5 s.

**Figure 13:**
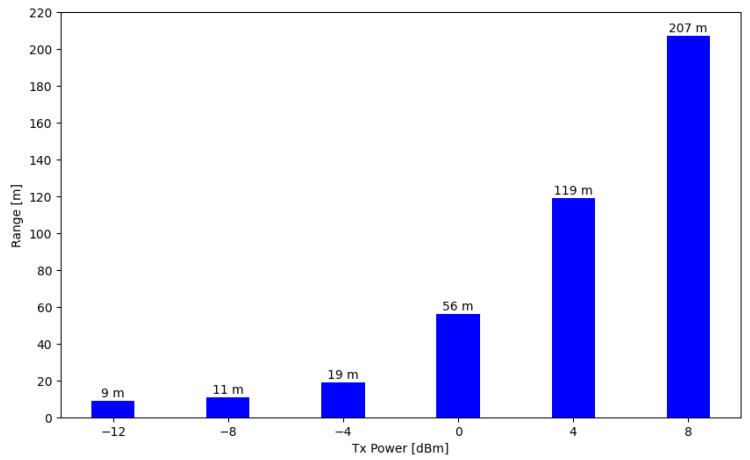
Relationship between communication range and TX power of the SN.

Transmission range tests were conducted using 3-byte advertising messages sent every second. The range was evaluated using different transmission powers of the SN. Although the SN typically operates with a transmission power of 0 dBm, its performance was assessed at various levels of BLE radio power, as shown in Figure 13.

### 5.4. Discussions

The experiments allowed comparing the energy consumption of the various SNs detailed in Table 1 with the SN used in this study. This comparison enables an assessment of related studies against the study conducted, as shown in Table 4.

To date, scientific literature lacks a practical implementation of the “FloatingBlue” architecture and does not report on its performance results. Although the study [44] offers the possibility of simulating the “FloatingBlue” architecture, it did not conduct specific tests simulating the combined use of DTN and BLE. The “FloatingBlue” proposal achieved success due to the software services developed in the application layer of the DM and the embedded system developed in the e-node. The graph in Figure 8 emphasizes optimizing the PDU message size to save energy in advertisement message transmissions.

One of the main challenges in WSNs is balancing energy consumption with communication range. Technologies that offer longer communication ranges tend to consume more energy, making applications impractical in scenarios where energy is a limited resource. In the case of “FloatingBlue”, tests have shown that increasing the BLE transmission power results in a significant increase in communication range, as illustrated in Figure 13. However, this extended range comes with a proportional increase in energy consumption. The trade-off between energy consumption and communication range is critical in situations where frequent battery replacement is impractical, and the FloatingBlue architecture can be applied, such as in wildlife monitoring, forest monitoring, precision agriculture, and disaster scenarios.

Therefore, it is essential to find a balance where the range is maximized without compromising the battery life of the sensor nodes. To achieve this balance, different levels of BLE transmission power and implementing intelligent energy management algorithms can be investigated to optimize the relationship between consumption and performance.

According to Table 4, the proposed beacon strategy for integration into the architecture stands out for having one of the lowest transmission consumption levels, attributed to the optimization of advertising messages and the bare-metal embedded software. In idle mode, favorable energy consumption is observed in the SN, which, despite being higher than those of studies using WuR technology [44], presents a significant advantage in terms of range. Thus, “FloatingBlue” offers lower transmission consumption and idle mode consumption similar to WuR, but with superior range, representing the main advancements compared to existing similar architectures.

## 6. Conclusions and Future Work

The performance of the “FloatingBlue” architecture has been evaluated as satisfactory, primarily due to its ability to transfer hundreds of packets from the ENs to the processing station in a remote and expansive scenario where there were long disconnect periods between the data mules. The proposed architecture demonstrated a high Packet Delivery Ratio (PDR) of 100%, ensuring reliable data transmission even in adverse conditions. Additionally, the optimizations implemented in the beacon’s energy consumption resulted in significant power savings, with 2.25 µJ in sleep mode and 11.8 µJ in transmission mode, surpassing the efficiency of other technologies, including RTOS-based solutions. These results highlight “FloatingBlue” as a highly energy-efficient solution for remote monitoring.

Furthermore, compared to related studies, the results regarding the beacon’s energy consumption in both idle and transmission modes were satisfactory, primarily attributed to optimizations implemented at the embedded software level.

Although the results obtained with “FloatingBlue” are promising, there are limitations that need to be addressed. Therefore, we recommend the following areas of investigation in future studies, corresponding to each identified limitation.

1. The requirement for line-of-sight conditions for efficient communication between sensor nodes and DMs, which can be compromised in scenarios with many obstacles, such as dense forests or rugged terrain. *Investigation:* incorporate LoRa radio technology into the physical layer of the DM to increase communication range;
2. Data security during transmission was not investigated, and future work could focus on implementing protocols to ensure data integrity and confidentiality. *Investigation:* developing an application-level security protocol to protect data transmitted by EN;
3. The dependency on batteries for the sensor nodes, as battery power can quickly deplete under conditions of high transmission frequency. *Investigation:* adopting a protocol that allows storage and transmission of messages from SN in multiple advertising packets.
4. The need for scalability testing with more SNs and DMs. *Investigation:* conduct studies through computer network simulations to analyze and verify the network’s behavior on a large scale.

## Figures and Tables

**Table 1:** Summary of related works.

Work	Year	End-Node Technology	DTN/OppNet	Sleep Mode	Tx Mode	Range (m)
[39]	2019	Zigbee	[ ]	50 µW	15 mW	7
[40]	2018	Zigbee	[✓]	181 mW	825 mW	100
[41]	2007	Zigbee	[✓]	80 mW	NA	1000
[7]	2020	LoRa	[✓]	72 µW	648 uW	NA
[6]	2020	LoRa	[✓]	231 mW	660 mW	NA
[8]	2018	LoRa	[✓]	24 mW	27 mW	NA
[42]	2022	IEEE 802.11	[ ]	9 µJ	41 µJ	25
[43]	2020	IEEE 802.11	[ ]	14 µW	494 µJ	NA
[44]	2011	Various	[✓]	633 nJ	100 mW	5
[17]	2018	BLE	[✓]	NA	NA	200
[24]	2017	BLE	[✓]	3.5 µJ	120 µJ	200
[45]	2016	BLE	[ ]	NA	57 mW	NA

**Table 2:** Comparative analysis of beacon power consumption: bare-metal firmware vs. RTOS firmware.

Firmware	Sleep Mode (µJ)	Tx Mode (µJ)
RTOS	13.4	24
Bare-Metal	2.25	11.8

**Table 3:** Configuration of dtn7.

Description	Parameter
Discovery Protocol	IPV4 e IPV6
Websocket	ws://0.0.0.0:8080/ws
CLA protocol	TCPCL
Routing Algorithm	Epidemic
Node-id	dtn://node-name/

**Table 4:** Comparative analysis of beacon power consumption: FloatingBlue vs. related works.

Work	Year	End-Node Technology	DTN/OppNet	Sleep Mode	Tx Mode	Range (m)
[39]	2019	Zigbee	[ ]	50 µW	15 mW	7
[40]	2018	Zigbee	[✓]	181 mW	825 mW	100
[41]	2007	Zigbee	[✓]	80 mW	NA	1000
[7]	2020	LoRa	[✓]	72 µW	648 µW	NA
[6]	2020	LoRa	[✓]	231 mW	660 mW	NA
[8]	2018	LoRa	[✓]	24 mW	27 mW	NA
[42]	2022	IEEE 802.11	[ ]	9 µJ	41 µJ	25
[43]	2020	IEEE 802.11	[ ]	14 µW	494 µJ	NA
[44]	2011	Various	[✓]	633 nJ	100 mW	5
[17]	2018	BLE	[✓]	NA	NA	~200
[24]	2017	BLE	[✓]	3.5 µJ	120 µJ	~200
[45]	2016	BLE	[ ]	NA	57 mW	NA
FloatingBlue	2024	BLE	[✓]	2.25 µJ	11.8 µJ	~200

## Data Availability

The data analyzed during the current study are available from the corresponding author on reasonable request.

## Abbreviations

The following abbreviations are used in this manuscript:

AA	Application Agent
BLE	Bluetooth Low Energy
CLA	Convergence Layer Adapter
DM	Data Mule
DTN	Delay Tolerant Networks
IEEE	Institute of Electrical and Electronics Engineers
IoT	Internet of Things
LDO	Low-Dropout
LPWAN	Low-Power Wide-Area Network
PDA	Public Device Addresses
PDR	Packet Delivery Ratio
PDU	Packet Data Unit
PPE	Personal Protective Equipment
RAM	Random Access Memory
RFC	Request for Comments
RTOS	Real-Time Operating System
SDK	Software Development Kit
SN	Sensor Node
SoC	System-on-a-Chip
TCPCL	Transmission Control Protocol Convergence-Layer
UAV	Unmanned Aerial Vehicles
WSNs	Wireless Sensor Networks
WuR	Wake-up Radio
